# Novel Antibody-Drug Conjugate with Anti-CD26 Humanized Monoclonal Antibody and Transcription Factor IIH (TFIIH) Inhibitor, Triptolide, Inhibits Tumor Growth via Impairing mRNA Synthesis

**DOI:** 10.3390/cancers11081138

**Published:** 2019-08-08

**Authors:** Mutsumi Hayashi, Hiroko Madokoro, Koji Yamada, Hiroko Nishida, Chikao Morimoto, Michiie Sakamoto, Hiroshi Yanagawa, Taketo Yamada

**Affiliations:** 1 Department of Pathology, Keio University School of Medicine, 35 Shinanomachi, Shinjuku-ku, Tokyo 160-8582, Japan; 2 Department of Pediatrics, Keio University School of Medicine, 35 Shinanomachi, Shinjuku-ku, Tokyo 160-8582, Japan; 3 Department of Biochemistry, The Jikei University School of Medicine, 3-25-8 Nishi-Shimbashi, Minato-ku, Tokyo 105-8461, Japan; 4 Department of Therapy Development and Innovation for Immune Disorders and Cancers, Juntendo University Graduate School of Medicine, 2-1-1 Hongo, Bunkyo-ku, Tokyo 113-8421, Japan; 5 Department of Fundamental Science and Technology, Keio University School of Science and Technology, 3-14-1 Hiyoshi, Kouhoku-ku, Yokohama City 233-8522, Kanagawa, Japan; 6 IDAC Theranostics Inc., 1-1-48 Suehirocho, Tsurumi-ku, Yokohama City 230-0045, Kanagawa, Japan; 7 Department of Pathology, Saitama Medical University, 38 Morohongo, Moroyama-machi, Saitama 350-0495, Japan

**Keywords:** antibody drug conjugate, targeted therapy, CD26, triptolide, RNA polymerase II, malignant mesothelioma

## Abstract

Here, we report a novel antibody drug conjugate (ADC) with the humanized anti-CD26 monoclonal antibody YS110 and triptolide (TR-1). YS110 has an inhibitory activity against the CD26-positive tumor growth via the immunological and direct pathway, such as intra-nuclear transportation of CD26 and YS110, and suppressed transcription of RNA polymerase II (Pol II) subunit POLR2A. The ADC conjugated with YS110 and an antitumor compound triptolide (TR-1), which is an inhibitor for TFIIH, one of the general transcription factors for Pol II was developed. YS110 and triptolide were crosslinked by the heterobifunctional linker succinimidyl 4-(N-maleimidomethyl)cyclohexane-1-carboxylate (SMCC) and designated Y-TR1. Antitumor efficacy of Y-TR1 against malignant mesothelioma and leukemia cell lines were assessed by the in vitro cell viability assay and in vivo assay using xenografted mouse models. Y-TR1 showed significant cytotoxicity against CD26-positive cell lines but not CD26-negative counterparts in a dose-dependent manner via suppression of mRNA synthesis by impairment of the Pol II activity. The tumors in xenografted mice administered Y-TR1 was smaller than that of the unconjugated YS110 treated mice without severe toxicity. In conclusion, the novel compound Y-TR1 showed antitumor properties against CD26-positive cancer cell lines both in vitro and in vivo without toxicity. The Y-TR1 is a unique antitumor ADC and functions against Pol II.

## 1. Introduction

CD26 is a type II glycoprotein that has intrinsic dipeptidyl peptidase IV (DPPIV) activity [1] and is implicated in broad and various physiological processes, including metabolism of glucose, activation of T lymphocytes, and cell adhesion [2,3]. CD26 has also been known as a cell surface marker associated with varied malignancies and as a part of cancer stem cells in mesothelioma and colon carcinoma [4,5,6]. Recent studies suggest that the expression of CD26 is functioned in tumor growth, tumor invasion, and metastasis [3,7,8].

We have already generated anti-CD26 monoclonal antibodies (mAbs) that have certain inhibitory effects against the growth of tumor cells and xenografted tumors [9,10]. This humanized anti-CD26 mAb YS110, that binds to the cell membrane-proximal glycosylated region starting at the 20-amino acid flexible stalk region of human CD26, has showed anti-tumor effects in malignant mesothelioma (MM) models [4]. Recently, the first-in-human phase 1/2 study of anti-CD26 mAbs in advanced cases with CD26-expressing mesothelioma and renal cell carcinoma has been done in France. As a result, anti-CD26 mAbs are well tolerated up to 6 mg/kg Q1W, which has been defined as RP2D, with encouraging prolonged disease stabilizations observed in a number of patients with advanced/refractory mesothelioma [11].

YS110 with a human IgG1 backbone recruits immune effector cells to human tumors, including natural killer (NK) cells, which express Fc receptors at the cell membrane, in antibody-dependent cellular cytotoxicity (ADCC). This Fc domain-based mechanism is observed with other therapeutic mAbs (e.g., trastuzumab and rituximab) commonly. These mAbs, approved for cancer therapy, also show direct inhibition of tumor growth. It has been reported that treatment with trastuzumab, a humanized anti-ErbB2 mAb, inhibits the cell growth of carcinoma cells by impairing a signaling pathway.

The nuclear localization of CD26 has been reported in cultured malignant mesothelioma and T cell leukemia lines and in human thyroid carcinomas [12]. The previous studies have shown that murine anti-CD26 mAb 1F7, which recognizes the identical epitope to YS110 and has anti-tumor effects against T cell lymphomas, induces internalization of CD26 and subsequently promotes its nuclear accumulation [12]. We have previously showed that the nuclear localization of CD26 is functionally involved in the anti-tumor process following the YS110 treatment and that the nuclear translocation of CD26 and YS110 contributes to growth inhibition of malignant mesothelioma cells after the YS110 treatment [13]. It was shown that the nuclear CD26 interacted with a specific genome target flanking the gene for the RNA polymerase II (Pol II) subunit POLR2A, which is indispensable for the transcription of almost genes, using chromatin immunoprecipitation (ChIP) cloning. This interaction between the flanking region of the POLR2A gene and CD26 led to the suppressed transcription of POLR2A mRNA. Furthermore, the impairing nuclear translocation of CD26 and YS110 prevented both the nuclear translocation of these two proteins and the YS110-induced transcriptional repression of the POLR2A gene [13]. These results reveal a novel function of CD26 as a transcriptional modulator in the nucleus and provide insight into the development of anti-cancer therapy through alteration of the nuclear translocation of cell-surface proteins.

**Figure 1:**
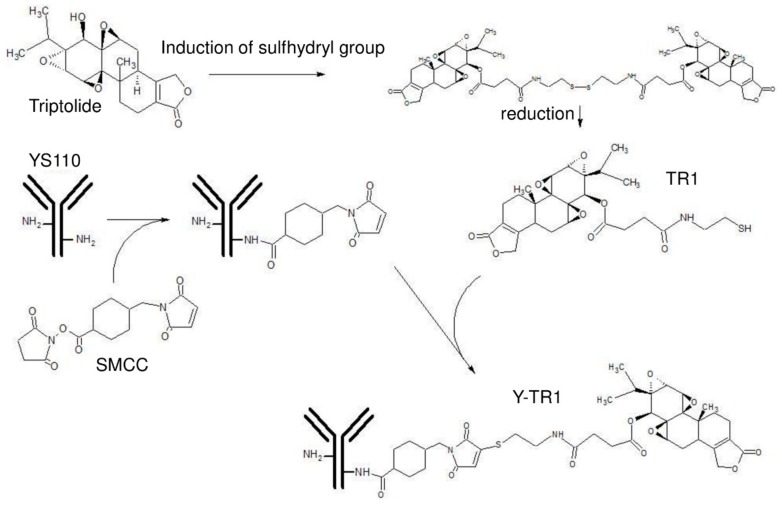
Structural formula of triptolide and conjugation protocol of Y-TR1 (SMCC). Triptolide was modified by a sulfhydryl (SH) group and was provided as an S-S dimer for chemical stability. The SH group-induced triptolide was designated TR1. The S-S bond was reduced to the SH monomer by the tris(2-carboxyethyl) phosphine (TCEP) reducing gel just before reaction. The humanized anti-CD26 monoclonal antibody YS110 was modified by the heterobifunctional linker SMCC. SMCC-modified YS110 and TR1 monomers were mixed and allowed to react overnight and purified. See Methods for details.

Herein, we designed a novel compound against CD26-positive cancers, which is constituted with YS110 and triptolide derivative TR-1 (Figure 1), an inhibitor for one of the general transcription factors for Pol II, TFIIH, and designated Y-TR1 [14]. It may be a reasonable strategy that the YS110 treatment specifically induces internalization of both CD26 and Y-TR1 into the nucleus from the cell surface in cancer cells but not in normal CD26-positive cells, such as the endothelium or lymphocytes, and then, YS110 suppresses the POLR2A transcription, and TR-1 inhibits TFIIH and POLII, which leads to additive or synergistic anti-tumor effects [15,16]. In this paper, it was revealed that Y-TR1 inhibits both cell growth of CD26-positive cancer cells and in vivo tumor growth in a xenograft model with CD26-positive tumor cells without toxicity.

## 2. Results

### 2.1. Cytotoxicity of Triptolide and TR-1 against MM and Leukemia Cell Lines

**Figure 2:**
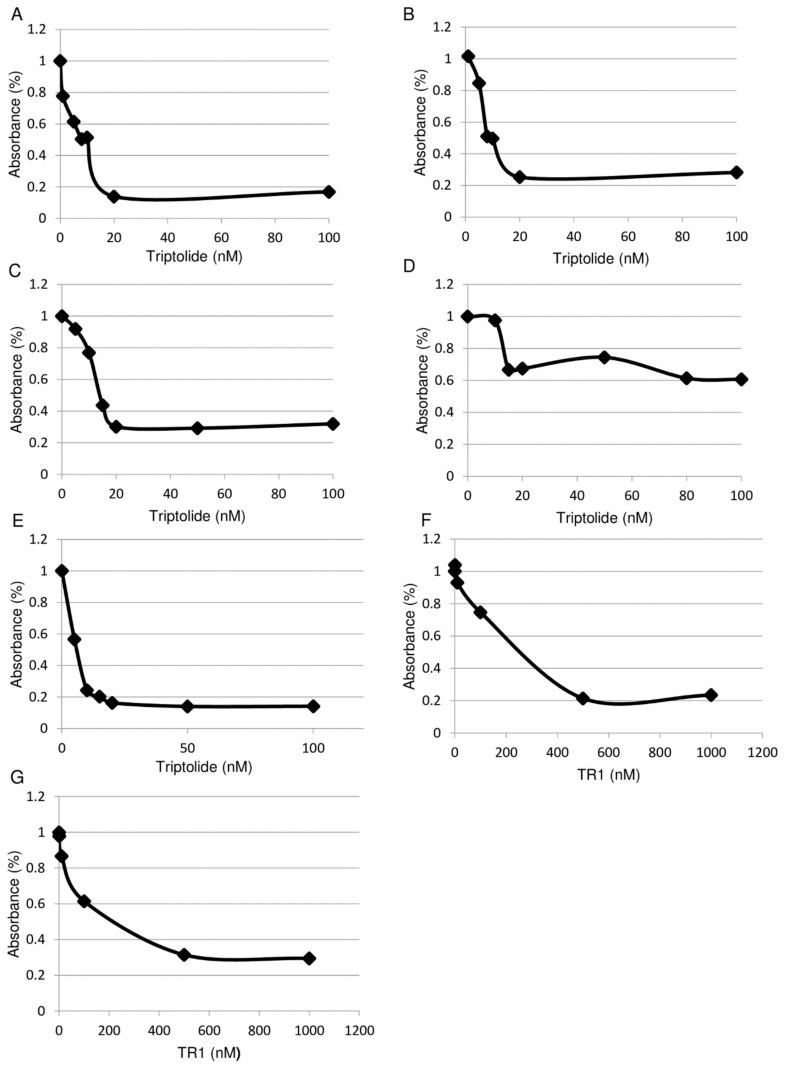
In vitro cytotoxic effect of Triptolide and TR1against MM cells and leukemia cells. Horizontal axis shows the concentration of the compounds in nM. Vertical axis shows the percent of control of the absorbance value in the WST-1 assay. The representative results of at least three independent experiments are shown. (**A**) Triptolide against MSTO wt; (**B**) triptolide against MSTO clone12; (**C**) triptolide against JMN; (**D**) triptolide against Jurkat (−); (**E**) triptolide against Jurkat CD26(+); (**F**) TR-1 against MSTO wt; (**G**) TR-1 against MSTO clone12.

As shown in Figure 2A–E, triptolide showed dose-dependent cytotoxic effects against the MM cell lines MSTO-wt, MSTO-clone12, JMN, and leukemia cell lines Jurkat, Jurkat CD26(+) after 48 h of treatment in WST-1 assays. The IC50 values of triptolide against these cell lines calculated from WST-1 assays are shown in Table 1.

Cytotoxicity and IC50 of triptolide derivative TR-1, designed for conjugation to the linkers, were tested in the same way and are shown in Figure 2F,G and Table 1. TR-1 was reduced by the immobilized tris(2-carboxyethyl) phosphine (TCEP) reducing gel (Thermo Scientific Inc., Waltham, MA, USA) from the S-S dimer before the assay.

### 2.2. Conjugation of YS110 and TR-1, the Drug Antibody Ratio of Y-TR, and the Binding Activity of Y-TR1 to CD26-Positive Cell Line

Conjugation of YS110 and TR-1 using heterobifunctional linkers (succinimidyl 3-(2-pyridyldithio)propionate (SPDP), N-γ-maleimidobutyryl-oxysuccinimide ester (GMBS), succinimidyl 4-(N-maleimidomethyl)cyclohexane-1-carboxylate (SMCC)) was performed following the procedures shown in Materials and Methods (Figure 1). The concentration of the final products measured by the BCA protein assay reagent kit (Thermo Scientific Inc.) was approximately 1 mg/mL. The remaining unconjugated TR1-SH in the product measured by the DTNB assay was almost undetectable. 

**Figure 3:**
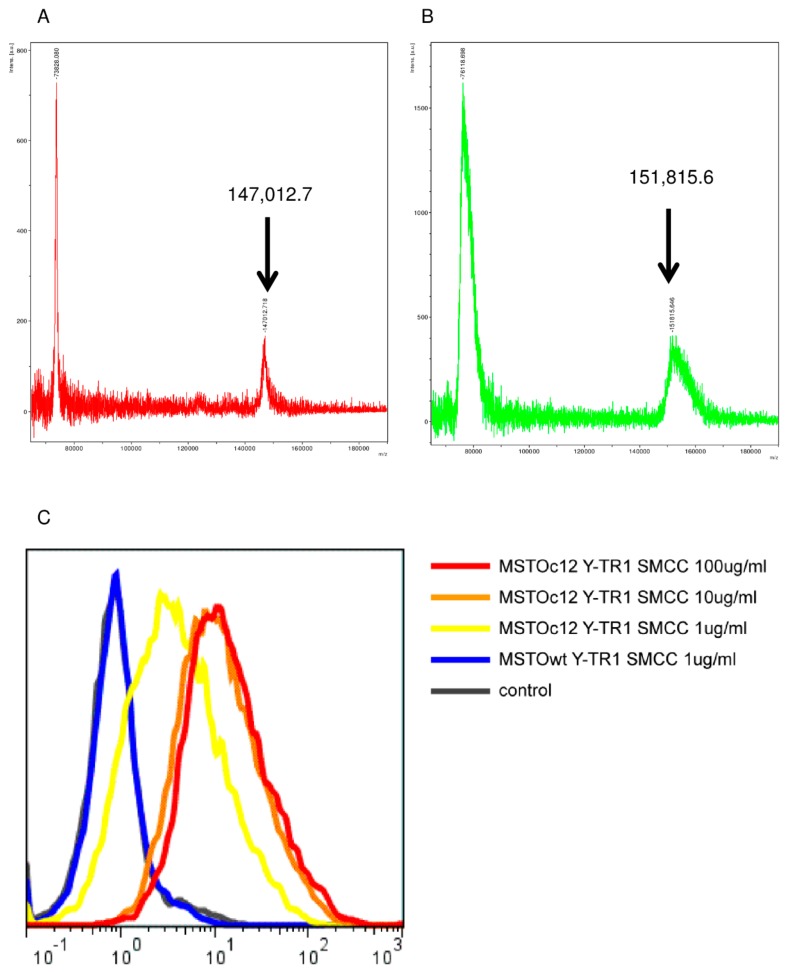
Biochemical analysis of Y-TR1. (**A**,**B**): The intact mass of unconjugated YS110 and Y-TR1 (SMCC) measured by the MALDI-TOF mass analysis. (**A**) Unconjugated YS110: 147,012.7; (**B**) Y-TR1 (SMCC): 151,815.6; (**C**) binding of Y-TR1 to the multiple myeloma (MM) cell line MSTO clone12 (CD26 positive). First antibody: Y-TR1 second antibody: Anti-human rabbit IgG FITC conjugate. Y-TR1 over 10 µg/mL showed intact binding to CD26-positive cells.

The intact masses of unconjugated YS110 and Y-TR1 (SMCC) measured by the MALDI-TOF mass analysis were 147,012.7 and 151,815.6, respectively (Figure 3A,B). The estimated molecular weight of a group of TR1 and SMCC conjugated with the antibody is 739.6. According to this molecular weight, the mean number of the TR1-SMCC groups conjugated with one molecule of YS110 is calculated by the calculation of (151,815.6−147,012.7) / 739.6, and the result is 6.489. This means 6.489 TR-1 molecules, on average, are conjugated with one molecule of YS110. 

The binding activity of Y-TR1 to the CD26-positive MM cell line MSTO clone12 was shown by the flow cytometry analysis (Figure 3C). 

### 2.3. In Vitro Cytotoxicity of Y-TR1 against MM and Leukemia Cell Lines

**Figure 4:**
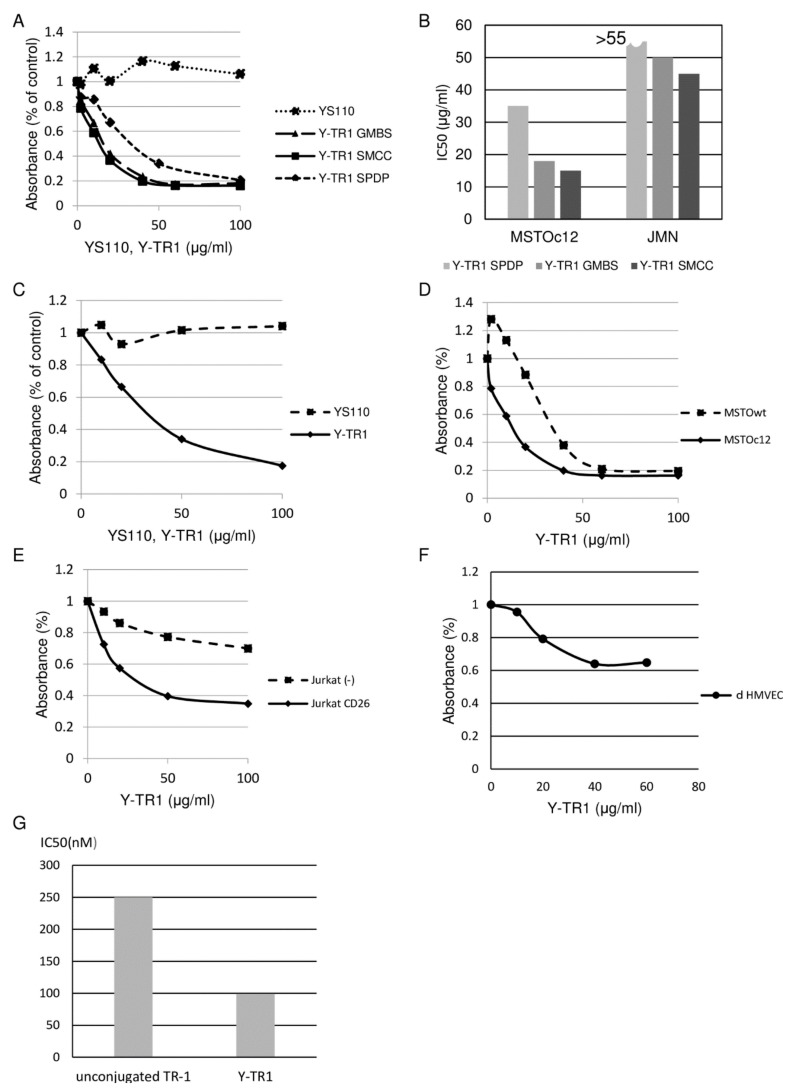
In vitro cytotoxicity of Y-TR1 against MM and leukemia cell lines. The representative results of at least three independent experiments are shown. (**A**) Comparison of cytotoxicity of Y-TR1 using various linkers against the CD26-positive MM cell line MSTO clone12. Y-TR1 using SMCC showed the highest cytotoxicity. The horizontal axis shows the concentration of the compounds in μg/mL. The vertical axis shows the percent of control of the absorbance value in the WST-1 assay; (**B**) comparison of IC50 of Y-TR1 using three linkers, SPDP, GMBS, and SMCC against CD26-positive MM cell lines MSTO clone12 and JMN; (**C**) in vitro cytotoxicity of TR-1 against the CD26-positive MM cell line JMN compared to unconjugated YS110. The horizontal and vertical axes show the same as indicated in (**A**); (**D** and **E**) higher in vitro cytotoxicity of Y-TR1 against the CD26-positive MM cell line MSTO clone12 compared to the CD26-negative counterpart MSTO wt (**D**) and CD26-positive leukemia cell line Jurkat CD26 (+) compared to the CD26-negative counterpart Jurkat (−) (**E**) at Y-TR1 concentration of 20 μg/mL. The vertical axis of the graph shows the percent of control in the WST-1 assay; (**F**) in vitro cytotoxicity of Y-TR1 against CD26-positive non-cancer adult dermal human microvascular endothelial cells (dHMVECs). Horizontal and vertical axes show the same as indicated in (**A**); (**G**) IC50 of unconjugated TR1 and Y-TR1 compared in molar concentration.

The Y-TR1 showed dose-dependent cytotoxicity against CD26-positive MM and leukemia cell lines (Figure 4A,C–E). We compared the Y-TR1 cytotoxicity against the CD26-positive MM cell line MSTO clone12 and JMN between three linkers, SPDP, GMBS, and SMCC (Figure 4A,B). Comparison between three heterobifunctional linkers was performed, and IC50 against the MSTO clone12 of Y-TR1 using SPDP, GMBS, and SMCC were 38 µg/mL, 18 µg/mL, and 15 µg/mL, respectively (Table 2). Since Y-TR1 conjugated by SMCC showed the best cytotoxicity in this experiment, we adopted Y-TR1 (SMCC) for further experiments. The cytotoxicity of Y-TR1 against various CD26-positive or -negative MM and leukemia cell lines was compared with unconjugated YS110 (Figure 4B). The IC50 values of Y-TR1 (SMCC) against various cell lines calculated from WST-1 assays are shown in Table 1. Against CD26-positive cells (MSTO clone12, JMN, Jurkat CD26 (+)), Y-TR1 showed remarkably higher cytotoxicity than the unconjugated YS110. Compared with the CD26 negative counterpart (MSTO wt, Jurkat CD26 (−)), CD26 positive cell lines (MSTO clone12, Jurkat CD26(+)) are more susceptible to the Y-TR1 cytotoxicity at the concentration of 20 μg/mL (Figure 4C,D). The in vitro influence of nonspecific binding of antibodies via the Fc receptor were evaluated by the cytotoxic assay of Y-TR1 with or without the Fc receptor blocking reagent. The Fc receptor blocking reagent had no significant effect on the in vitro cytotoxicity of Y-TR1 against MSTO clone12 cells (Supplementary Figure S1). In vitro cytotoxicity against CD26-positive non-cancer cells (primary dermal human microvascular endothelial cells, dHMVECs) was also tested, and Y-TR1 showed less cytotoxicity than against CD26-positive malignant cell lines significantly (Figure 4E). 40% reduction in the viability of dHMVECs was observed at the maximum concentration of Y-TR1 (60 µg/mL).

Y-TR1’s equivalent of free TR-1 was calculated by IC50 of Y-TR1 (approximately 15 µg/mL = 99 nM) and unconjugated TR-1 (250 nM) against the MSTO clone12 cell line. The calculated value was approximately 2.5 Y-TR1 molecules equivalent (Figure 4F).

### 2.4. Nucler Translocation of Y-TR1

**Figure 5:**
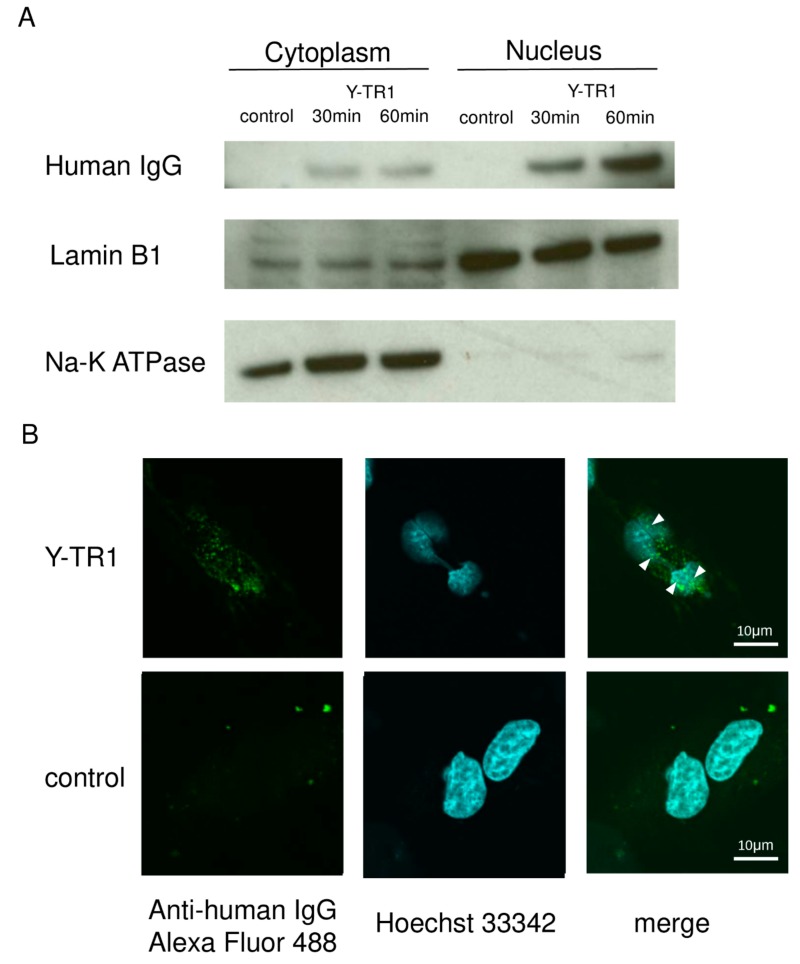
Nuclear translocation of Y-TR1. (**A**) Western blot analysis using anti-human IgG antibody detected Y-TR1 in the cytoplasm and the nuclear fraction of Y-TR1 treated CD26 positive JMN cells after 30 min and 60 min. Lamin B1 and Na-K ATPase were used as loading controls of the nuclear and cytoplasm fraction; (**B**) immunofluorescence staining observed under confocal laser microscopy of fixed JMN cells following 1 h of Y-TR1 treatment with the Alexa Fluor 488 labeled anti-human IgG antibody. Nuclear staining was done with Hoechst 33342. Localization of Y-TR1 (green) was observed in the nucleus (blue) as indicated by the white arrows. Scale bar: 10μm.

To confirm that Y-TR1 molecules are internalized and are transported into the nucleus in CD26 positive cancer cell lines as with YS110, the Western blot analysis of nuclear fraction using the anti-human IgG antibody and immunofluorescence staining using were conducted. In the western blot analysis of the CD26 positive JMN cell line, Y-TR1 molecules were detected by the Western blot analysis using the anti-human IgG antibody in both the cytoplasm and nuclear fraction after 30 min and 60 min of the Y-TR1 treatment (Figure 5A). Immunofluorescence staining using the Alexa Fluor 488 labeled anti-human IgG antibody observed under confocal laser microscope showed several dots colocalized with nuclear staining (Hoechst 33342) in Y-TR1 treated (60 min) JMN cells (Figure 5B).

### 2.5. Apoptosis Assay

The induction of apoptosis was examined after the Y-TR1 treatment because triptolide has been reported to cause apoptosis in cancer cells [17]. After 48 h of the Y-TR1 treatment, the caspase 3/7 activity of triptolide-, TR1-, and Y-TR1-treated MSTO clone12 cells was elevated six to nine times against YS110-treated cells (Figure 5A). These results support the estimation that the cytotoxic effects of Y-TR1 are caused by internalized TR1.

### 2.6. Effects of Triptolide and Y-TR1 on Heat Induction of HSP70 in CD26-Positive MM Cells

To confirm that the cytotoxic effect of Y-TR1 is caused by RNA polymerase II repression as with triptolide, heat shock induction of HSP70, which is dependent on the RNA polymerase activity, was evaluated [17]. In both CD26-positive MM cell lines MSTO clone12 and JMN, the mRNA level of HSP70 after heat shock (45 °C, 2 h) in Y-TR1-treated cells was significantly lower than in YS110-treated cells significantly (Figure 5B,C).

### 2.7. In Vivo Anti-Tumor Effect of Y-TR1

**Figure 6:**
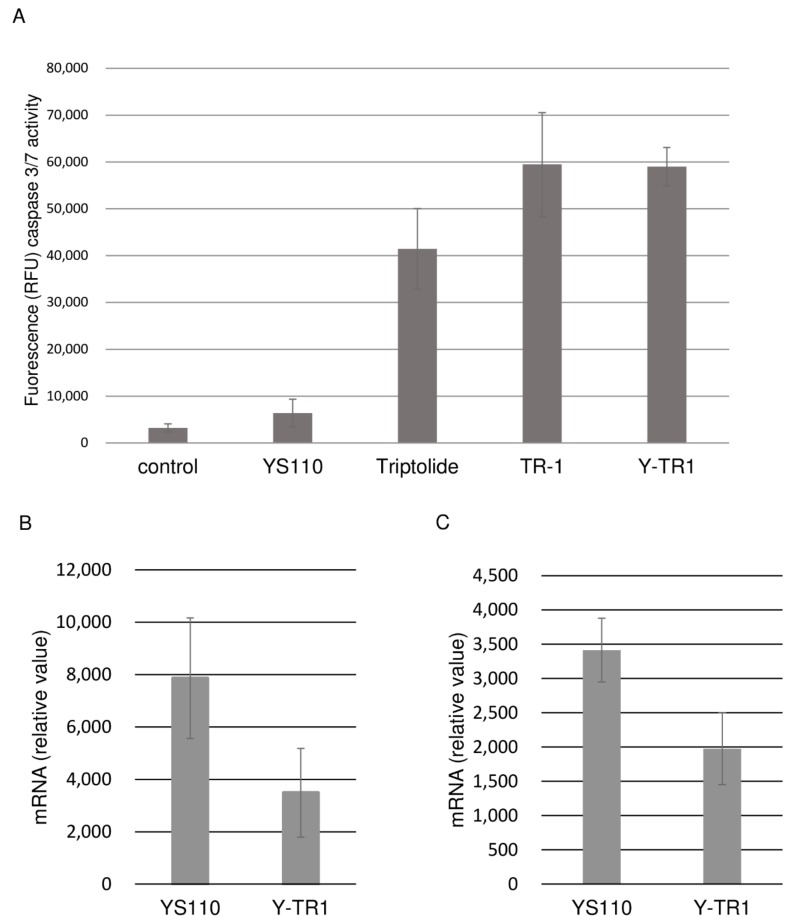
Investigation into the cytotoxic effect of TR-1. (**A**) Caspase 3/7 activity (represented in fluorescence) after 48 h of treatment with YS110, triptolide, TR1, and Y-TR1 in the CD26-positive MM cell line MSTO clone12. The activity is elevated in triptolide-, TR1-, and Y-TR1-treated cells. The vertical axis shows the intensity value measured by the fluorometer; (**B**, **C**) effects of Y-TR1 on the mRNA levels of HSP70 after heat induction. Relative mRNA levels of HSP70 after heat induction (45 °C, 2 h) were significantly lower in Y-TR1-treated cells compared to unconjugated YS110-treated cells in CD26-positive MM cell lines. A *t*-test at the *p* = 0.05 level was carried out as statistical analysis (*n* = 10). The error bar indicates one standard deviation; (**B**) MSTO clone12; (**C**) JMN.

The in vivo efficacy of Y-TR1 compared with the unconjugated YS110 in the NOD/SCID mouse xenograft model using the CD26-positive MM cell line JMN. The mean tumor volumes on day 55 estimated by the ellipsoid volume formula (π/6 × L × W × H) [18] were compared between three groups (control, YS110, Y-TR1, *n* = 10) with Fisher’s protected least protected difference multiple comparison test. The mean tumor volume of the Y-TR1 group (4 mg/kg weight, total 36 mg/kg Y-TR1 intraperitoneally) was significantly lower (*p* < 0.05) than the control or the YS110 group (Figure 6A). The mean tumor volume of the YS110 group (4 mg/kg weight, total 36 mg/kg Y-TR1 intraperitoneally) was not significantly altered compared with the control (Figure 6A). Two of the nine Y-TR1 group mice showed complete tumor growth prevention macroscopically at the time of sacrifice (day 55). One representative experiment out of two with similar results is shown. There were no clinical manifestations in mice treated with YS110 or Y-TR1. The mean body weight of the mice of each group at sacrifice was not significantly different in mice treated with YS110 or Y-TR1 (Supplementary Figure S2A). No pathological alterations were observed in the brain, heart, lung, liver, spleen, kidney, pancreas, digestive organs or adrenal glands of mice (Supplementary Figure S2B). On the other hand, the mean tumor weight of the YS110 only group and the Y-TR1 group (8 mg/kg weight, total 72 mg/kg of YS110 or Y-TR1 intraperitoneally) was significantly lower (*p* < 0.05 or *p* < 0.025) than that of the control group (Figure 6B). Furthermore, the mean tumor weight of the Y-TR1 group was significantly lower (*p* < 0.05) than that of the YS110 only group (Figure 6B). The statistical analyses were done using Fisher’s protected least protected difference multiple comparison test (*n* = 10). In these tumors, the cell growth analysis was performed using MIB-1 (Ki67) staining. As a result, a decreased number of MIB-1-positive cells in Y-TR1-treated tumors was shown compared to IgG1- or YS110-treated tumors (Figure 6C).

## 3. Discussion

RNA polymerase II is indispensable for the transcription of almost all protein-coding genes, including those related to cell proliferation [19]. It was previously reported that the functional blockade of one of the subunits of RNA polymerase II, POLR2A, by RNAi strategies and treatment with chemical compounds such as α-amanitin resulted in growth inhibition of cancer cells [20,21,22]. We have shown that nuclear accumulation of CD26 promoted POLR2A suppression, leading to a reduction in cell growth [13]. Therefore, we examined whether the novel Antibody-drug conjugate (ADC), Y-TR1 (YS110-TR1 conjugate), restrained POLR2A expression more strongly in the nucleus of tumor cells than YS110 alone. Triptolide has been demonstrated to possess a unique bioactive spectrum of anti-cancer activity and immunosuppressive efficacy; however, due to its poor water solubility and severe toxicity, triptolide cannot be used systemically in the clinic [14]. Therefore, we attempted to develop a new ADC, Y-TR1, which has a higher efficiency of anti-tumor action because triptolide was conjugated with humanized anti-human CD26 monoclonal antibody, YS110, with anti-tumor effects via the suppression of POLR2A transcription, retarded G2/M cell cycling, and antibody-dependent cell-mediated cytotoxicity (ADCC) / complement-dependent cytotoxicity (CDC) [13,23]. 

Recently, Liu Y et al. reported that cancer cells with hemizygous TP53 deletion were vulnerable to further suppression of such genes. POLR2A was identified as a gene that is almost always co-deleted with TP53 in human cancers [20]. Suppression of POLR2A with α-amanitin or small interfering RNAs selectively inhibits the proliferation, survival and tumorigenic potential of colorectal cancer cells with hemizygous TP53 loss in a p53-independent manner [24]. However, some previous clinical applications of POLR2A inhibitors, such as α-amanitin, have been limited due to their liver toxicity [25]. Therefore, they suggested that α-amanitin-based antibody–drug conjugates were highly effective therapeutic agents with reduced toxicity [26]. It was shown that low doses of the α-amanitin-conjugated anti-epithelial cell adhesion molecule (EpCAM) antibody lead to complete tumor regression in mouse models of human colorectal cancer with hemizygous deletion of POLR2A [24]. TP53 is frequently inactivated in mesothelioma, but mutations are rare. MDM2 and P14/ARF are upstream regulators of TP53 that may contribute to TP53 inactivation [27]. These results suggest that POLR2A may be a certain therapeutic target molecule for malignancies.

Most therapeutic mAbs are thought to disturb signal transduction within tumor cells or get rid of critical cell-surface antigens [28]. As a consequence, these effects may lead to the clearance of cancer cells. ErbB2 is known to be associated with a specific locus on the cyclooxygenase (COX) 2 promoter, activate the gene expressions, thereby inducing cell growth [29]. The humanized ErbB2 mAb trastuzumab inhibits the translocation of ErbB2 into the nucleus. Herein it revealed that in contrast to this ErbB2-Herceptin line, the YS110 treatment abundantly induces nuclear localization of CD26 and in consequentially suppresses POLR2A expression, leading to inhibition of tumor cell growth. These findings reveal that disturbance of nuclear transport of cell-surface antigens by mAbs may be effective targets for mAb therapy against malignancies. Recently it has be shown that some mAbs conjugated to payloads (e.g., radioisotopes, drugs, or toxins) may be targeted to direct inductions of tumor cell death [30,31,32,33]. The 90Y-radiolabeled anti-CD20 IgG1, Ibritumomab tiuxetan, has been examined to have substantial anti-tumor effects and is available for standard clinical practice as a therapy for lymphoma [34]. However, the potent cytotoxicity of these payloads may delay the development of novel conjugated antibodies. We have revealed the nuclear localization of anti-CD26 mAbs YS110 in a cell-surface CD26-dependent manner. This phenomenon implies that YS110 may be a target for specific intra-nuclear components, such as genomic DNA sequences and transcription factors. There have been previous reports on the nuclear localization of mAbs against cell-surface antigens, such as ME425 (against EGF receptor) and Br 15-6A (against carbohydrate Y determinant) [35,36]. 

As the clinical application of ADCs advances, difficulties in the drug-antibody ratio (DAR) control has been discussed recently. FDA approved ADC Kadcyla (T-DM1) is conjugated with the SMCC linker using the amino side chains of lysine residue of the antibody as with Y-TR1. As the limit of the procedure, the ADCs are a heterogenous mixture of ADCs with several DARs [37]. Though some of the efforts to develop methodologies to obtain homogenous ADCs using site specific conjugation have been proposed, many of these technologies require additional bio-engineering or chemical work and not fully established [37]. At the moment, we concluded that the conventional method using the SMCC linker is the best option considering accumulated data of Kadcyla and Adcetris (Brentuximab vedotin). 

**Figure 7:**
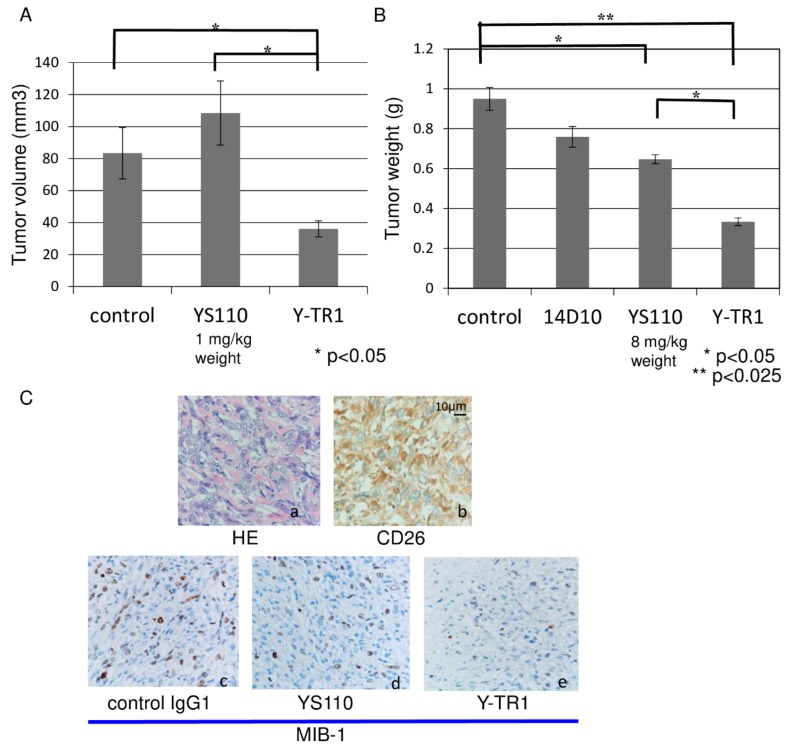
In vivo anti-tumor effect of Y-TR1 in the NOD/SCID mouse xenograft model using the CD26 positive MM cell line JMN. (**A**) Y-TR1 was administered intraperitoneally 4 mg/kg/dose, three times per week, for a total of nine doses from day zero of subcutaneous inoculation of 1 × 107 JMN cells. The average estimated tumor volume on day 55 was compared among three groups (control, YS110, Y-TR1, *n* = 10) with Fisher’s protected least protected difference multiple comparison test. The mean tumor volume of the Y-TR1 group was significantly lower (* *p* < 0.05) than that of the control or YS110 group. The mean tumor volume of the YS110 group was not significantly altered compared with the control. An experiment out of two with similar results is shown; (**B**) Y-TR1 was administered intraperitoneally 8 mg/kg/dose, three times per week, for a total of nine doses. The average estimated tumor weight on day 42 was compared among three groups (control, 14D10, YS110, Y-TR1, *n* = 10) with Fisher’s protected least protected difference multiple comparison test. The mean tumor weight of the YS110 or Y-TR1 groups was significantly lower (* *p* < 0.05 or ** *p* < 0.025, respectively) than that of the control group. The mean tumor weight of the Y-TR1 group was significantly lower (* *p* < 0.05) than that of the YS110 group. An experiment out of two with similar results is shown; (**C**) histological analysis of xenograft tumors of JMN cells. JMN-derived tumors show histopathology of sarcomatoid mesothelioma. (×20). a: Hematoxylin and eosin staining. b: Immunohistochemical staining with anti-human CD26 antibody revealed CD26 expression in tumor cells. c–e: MIB-1 (Ki67) staining showed a decreased number of MIB-1-positive cells in Y-TR1-treated tumors compared to IgG1- or YS110-treated tumors. Scale bar: 10 μm.

**Figure 8:**
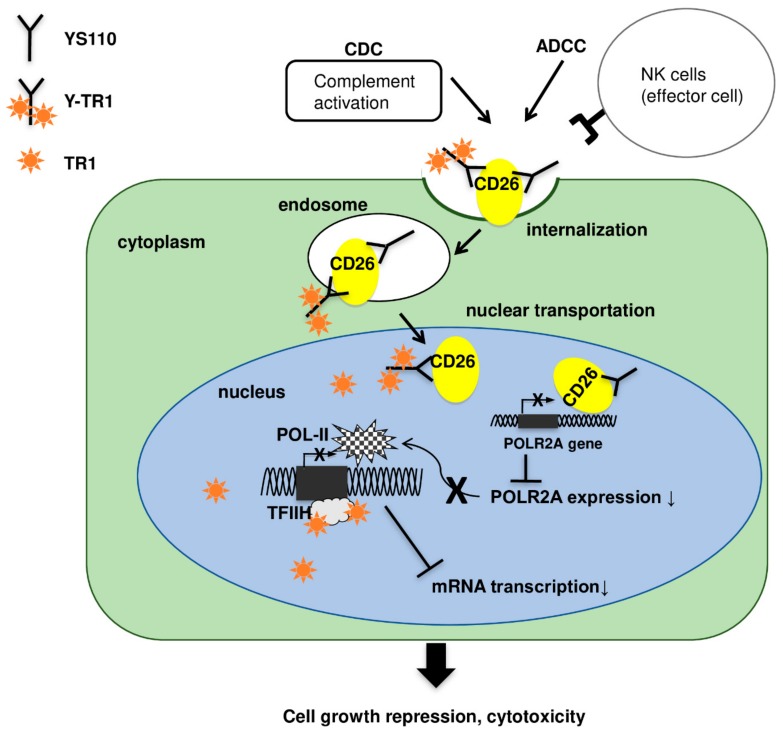
Y-TR1 has multiple anti-tumor effects as follows; (1) introduction of cell death via immunological cytotoxicity such as antibody-dependent cell-mediated cytotoxicity (ADCC) and/or complement-dependent cytotoxicity (CDC), (2) retarded cell cycling of both G1/S and G2/M, (3) suppression of POLR2A transcription by increased amount of intranuclear CD26, (4) inhibition of TFIIH by TR1 carried into the nucleus using conjugation of TR1 to YS110.

The overview of anti-cancer effects of antibody drug conjugate Y-TR1 was shown in Figure 7 and Figure 8. It is expected that Y-TR1 bound to CD26 on the cell surface introduces cell death via immunological cytotoxicity such as ADCC and/or CDC [4,11]. On the other hand, CD26 and Y-TR1 are internalized into cytoplasm and then transported to the nucleus within 1 h as confirmed in this study by the immunofluorescence study and Western blot study. In the nucleus, the suppression of POLR2A transcription by the increased amount of intra-nuclear CD26 and the inhibition of TFIIH by TR1 impairs mRNA synthesis [12,13,16], as indicated in this study. Furthermore, YS110 retards directly cell cycling of cancer cells at both G1/s and G2/M [9,10,23].

The LD50 of triptolide was reported as 0.83 mg/kg body weight in mice [38]. The calculated LD50 of TR-1, triptolide-derivative for conjugation to YS110, is 20.8 mg/kg body weight because the TR-1 has reduced anti-tumor activity to 1/25 of triptolide IC50. So, the LD50 of Y-TR1 may be calculated as 932.5 mg/kg body weight in the condition that all TR1 conjugated with YS110 was released from ADC, Y-TR1, because Y-TR1 has 6–7 molecules of TR1 on one molecule of Y-TR1. As a result, the clinical application of Y-TR1 may be expected at 6 mg/kg body weight in accord with the concentration of YS110 in the phase I clinical trial without toxicity of TR1 because the LD50 of Y-TR1 is estranged from the calculated LD50 of TR-1.

In consequence, the present data show certain evidences that induced that the nuclear localization of CD26 by the humanized anti-CD26 mAb YS110 promotes transcriptional repression of the POLR2A gene, and then, the internalization of YS110-TR-1 compound into the nucleus may inhibit TFIIH, resulting in growth suppression of cancer cells. Given that Y-TR-1 has a direct anti-proliferative effect on cancer cells, including malignant mesothelioma cells, these findings highlight the potential of rational therapy against CD26-positive cancers, not only through immunological ADCC and complementary activation effects but also by direct inhibition of cancer cell growth.

## 4. Materials and Methods 

### 4.1. Reagents and Antibodies

The humanized anti-CD26 antibody YS110 was constructed from the anti-CD26 mouse monoclonal antibody 14D10 coding sequence as described previously [4]. Triptolide (Figure 1) was purchased from Shaanxi Taiji Huaqing Technology (Shaanxi, China), and an SH group was introduced by ChemGenesis Inc. (Tokyo, Japan). The triptolide derivative was designated TR1. TR-1 was provided as an S-S dimer for chemical stability (Figure 1).

### 4.2. Conjugation Protocols

Heterobifunctional linkers, SPDP (N-Succinimidyl 3-(2-pyridyldithio)-propionate) (Cat No. 21857, Thermo Scientific Inc.), GMBS (N-[γ-maleimidobutyryloxy]succinimide ester) (Cat No. 22309, Thermo Scientific Inc.), SMCC (succinimidyl 4-[N-maleimidomethyl] cyclohexane-1-carboxylate) (Cat No. 22360, Thermo Scientific Inc.) were solved in dimethyl sulfoxide (DMSO) just before use. YS110 was modified with heterobifunctional linkers SPDP (15 times molar excess), GMBS (30 times molar excess), or SMCC (20 times molar excess) in PBS-EDTA pH 7.5 at room temperature for 30 min. The unreacted excess linkers were removed by the HiTrap Desalting Column (Cat No. Cat No. 21857, GE Healthcare Inc., Buckinghamshire, UK). The triptolide derivative TR1 S-S dimer was resolved in 100% ethanol and reduced using the immobilized TCEP reducing gel (Cat No. 77712, Thermo Scientific Inc.) for 2 h. The concentration of the SH group of reduced TR1-SH was measured by the DTNB ((5,5-dithio-bis-(2-nitrobenzoic acid)) assay. Linker-modified YS110 and TR-1-SH, at a ratio of 1:5.68, were reacted in PBS-EDTA pH 7.5 at room temperature overnight. Unreacted TR-1-SH was removed by the PD-10 Column (Cat No. 17085101, GE Healthcare Inc.). The product was sterilized by filtration with the Millex-GV Filter Unit 0.22 μm (Cat No. SLGV 013SL, EMD Millipore, Billerica, MA, USA), and the final concentration was measured using a BCA protein assay reagent kit (Cat No. 23225, Thermo Scientific Inc.). The outline figure of the conjugation protocol is indicated in Figure 1. The remaining unconjugated TR1-SH in the product was measured by the DTNB assay.

### 4.3. Cell Culture 

MSTO-211H (MSTO) (American Type Cell Culture Collection, Manassas, VA, USA), a CD26-negative malignant mesothelioma cell line, was transfected with the CD26 gene and designated MSTO-clone12 [13]. Jurkat (American Type Cell Culture Collection), CD26 negative T-cell leukemia cell line, was transfected with the CD26 gene and designated Jurkat CD26(+) [39]. JMN, a CD26-positive cell line established from malignant mesothelioma, was provided by the Clinical Research Center, Institute of Medical Science, University of Tokyo. All the cell lines were grown in the RPMI medium (Cat No. 11875-093, Life Technologies, Carlsbad, CA, USA) supplemented with 10% heat-inactivated fetal bovine serum (FBS, Life Technologies), ABPC (100 µg/mL), Streptomycin (100 µg/mL), 37 °C, 5% CO_2_. dHMVEC (American Type Cell Culture Collection), primary dermal human microvascular endothelial cells were grown in the EGM-2MV Bullet Kit medium (Lonza, Basel, Switzerland) at 37 °C, 5% CO_2_.

### 4.4. Mass Spectrometry Assay

The drug-antibody ratio of Y-TR1 was analyzed by the Matrix Assisted Laser Desorption/Ionization Time of Flight Mass Spectrometry (MALDI-TOF mass) using Autoflex III (Bruker Corporation, Billerica, MA, USA) after ultrafiltration.

### 4.5. Binding Assay

To assess the binding of Y-TR1 to the CD26-positive MM cells, cultured MSTO-wt (CD26 negative) and MSTO-clone12 (CD26 positive) cells were collected, and 1 × 106 cells were incubated with 1 µg/mL, 10 µg/mL, and 100 µg/mL of Y-TR1 at 4 °C for 30 min. Cells were washed three times and incubated with FITC-conjugated rabbit anti-human IgG (Cat. No. 6140-02, Southern Biotech, Birmingham, AL, USA) at a 1:100 dilution at 4 °C for 30 min. After washing three times, the FACS analysis was carried out on Epics XL-MCL (Beckman Coulter, Brea, CA, USA).

### 4.6. Cytotoxicity Assay

The cytotoxic effects of triptolide, TR-1, YS110 and Y-TR1 against MM and T-cell leukemia cell lines were measured using the colorimetric cell proliferation kit WST-1 (Cat No. 11644807001, Roche Applied Science, Rotkreuz, Switzerland) based on the colorimetric detection of a formazan salt. In brief, 5 × 103 MSTO-wt, MSTO-clone12, JMN, Jurkat CD26(−), and Jurkat CD26(+) cultured in 96-well plates in the RPMI-1640 medium supplemented with 10% heat-inactivated fetal bovine serum (FBS), ABPC(100 μg/mL), Streptomycin(100 μg/mL) with Triptolide (ranging from 0 nM to 100 nM), TR-1 (ranging from 0 nM to 1000 nM), YS110 (ranging from 0 µg/mL to 100 µg/mL), or Y-TR1 (ranging from 0 µg/mL to 100 µg/mL) for 48 h at 37 °C, 5% CO_2_. To assess the influence of nonspecific binding of Y-TR1 to Fc receptors, the cytotoxicity assay of Y-TR1 against MSTO-clone12 cells carried out as above with 2 μg/mL of the Fc receptor blocking reagent, Human BD Fc Block (Cat.No. 564219, BD Life Sciences, Franklin Lakes, NJ). CD26-positive dHMVECs cultured in the EGM-2MV Bullet Kit medium (Lonza) underwent the same procedure as above. The WST-1 assay was carried out according to the manufacturer’s instructions. The background absorbance of each sample at 630 nm was subtracted from the readings at 450 nm. The experiment was performed in triplicate, and the representative experiment is shown.

### 4.7. Western Blotting

Cultured CD26 positive MM cell line JMN cells were treated for 30 min or 60 min with Y-TR1 (2 μg/mL). Nuclear and cytoplasmic protein fractions were obtained using the NE-PER Nuclear and cytoplasmic extraction reagents (Cat No. 78833, Thermo Fischer Scientific Inc.) following the manufacturer’s instructions. For the Western blot analysis, 20 μg of cytoplasmic fraction and 5 μg of nuclear protein were separated on an SDS-polyacrylamide gel and transferred to a PVDF membrane by the Trans-Blot Turbo Transfer System (Bio-Rad Laboratories, Hercules, CA, USA). After blocking for 1 h in the Bullet Blocking One for Western Blotting reagent (Cat No. 13779-01, nacalai tesque, Kyoto, Japan), the membrane was incubated with Rabbit F(ab’)2 Anti-Human IgG (H+L)-HRP antibody (Cat No. 6000-05, Southern Biotech) diluted 1:1000 in Can Get Signal Solution 2 (Cat. No. NKB-101T, TOYOBO, Osaka, Japan) for 30 min at room temperature and developed using the ECL Western Blotting Detection Reagents (GE Healthcare, Buckinghamshire, UK). The anti-Lamin B1 antibody (Cat. No. sc-6216, Santa Cruz Biotechnology, Dallas, TX, USA) and Anti-Na-K ATPase α1 antibody (Cat. No. sc-21712, Santa Cruz Biotechnology) were used as loading controls for the nuclear and membrane/cytoplasm fraction, respectively. The experiment was performed in triplicate, and the representative experiment is shown.

### 4.8. Immunofluorescence Staining

CD26 positive MM cell line JMN cells cultured on chamber slides were treated with Y-TR1 (2 μg/mL) for 60 min. PBS (phosphate buffered saline) were added to the control cells. The cells were fixed in 4% paraformaldehyde for 15 min and permeabilized by 0.1% TritonX-100 for 10 min at room temperature. The cells were incubated with Rabbit F(ab’)2 anti-human IgG (H+L)-Alexa Fluor 488 (Cat. No. 6000-05, Southern Biotech) diluted 1:100 in Can Get Signal Solution 1 (Cat. No. NKB-101T, TOYOBO) for 60 min at room temperature. Nuclear staining was done with Hoechst 33342 (Cat. No. H3570, Thermo Scientific Inc.) diluted 1:2000 in 1% BSA for 10 min at room temperature. Stained cells were examined by the confocal laser microscopy FV10i (Olympus, Tokyo, Japan).

### 4.9. Apoptosis Assay

The CD26-positive MM cell line MSTO-clone12 was treated with YS110 (40 µg/mL), triptolide (20 nM), TR-1 (400 nM), and Y-TR1 (40 µg/mL) for 48 h. After 48 h of treatment, the groups of cells underwent apoptosis assays using the Apo-ONE Homogenous Caspase 3/7 assay (Cat. No. G7792, Promega Corporation, Madison, WI, USA). The assays were performed according to the manufacturer’s instructions. In short, active caspase 3/7 in the lysed cells catalyzes profluorescent substrate tofluorescent product. The intensity of the fluorescence was measured using a fluorometer (Glomax Multi Detection System, Promega Corporation). The experiment was performed in triplicate, and the representative experiment is shown.

### 4.10. Heat Induction of HSP70 and Real Time PCR Assay

CD26-positive MM cell lines MSTO-clone12 and JMN were treated with unconjugated YS110 (40 µg/mL) or Y-TR1 (40 µg/mL) for 1 h before heat shock (45 °C, 2 h). After heat shock, the cells were lysed immediately to isolate the total RNA using an RNeasy mini kit (Cat. No. 74104, Qiagen, Hilden, Germany). Total RNA was reverse transcribed using Prime Script RT enzyme (Takara Bio Inc., Shiga, Japan), and cDNA was used for real-time PCR with the following primers. HSP70 (forward): CAC CAC CTA CTC CGA CAA CCA, HSP70 (reverse): GCG CCT AAT CTA CCT CCT CAA TG, (Invitrogen, Carlsbad, CA) beta actin (forward): TGG CAC CCA GCA CAA TGA A, beta actin (reverse): CTA AGT CAT AGT CCG CCT AGA AGC A (Takara Bio inc., Shiga, Japan). Real-time PCR reactions were performed using Thermal Cycler Dice TP800 (Takara Bio Inc.). The experiment was performed in triplicate, and the representative experiment is shown.

### 4.11. In Vivo Efficacy Assay and Toxicity Study

NOD/SCID (NOD/LtSz-scid) mice were maintained in a specific pathogen-free facility in micro-isolator cages and were provided with sterile food and water ad libitum. The animal protocol was approved by the Keio University Institutional Animal Care and Use Committee (approval number: 9184). A total of 1×107 cultured JMN cells were subcutaneously transplanted into female 6- to 8-week-old NOD/SCID mice. All 30 animals were randomly assigned into four treatment groups, i.e., control, YS110, 14D10 and Y-TR1 groups. From the day of transplantation, the YS110, 14D10 and Y-TR1 groups received 4 or 8 mg/kg/dose of YS110, 14D10 or Y-TR1 intraperitoneally three times a week, for a total of nine doses. The control group received an equivalent volume of human IgG1 (Sigma-Aldrich, Tokyo, Japan). When the tumor became apparently visible, the tumor was excised and measured by caliper and weighed. The estimated tumor volume was calculated by the formula of π/6 × L × W × W [18]. One representative experiment out of two with similar results is shown. Tumor tissues were fixed in 10% neutral buffered formalin, embedded in paraffin, and sectioned at a thickness of 5 µm. For histology, sections were stained with hematoxylin and eosin. For immunohistochemistry, sections were washed with PBS and subjected to antigen retrieval by heating at 100 °C in 0.01 M sodium citrate (pH 6.0) for 10 min and then treated with 3% H_2_O_2_ before incubation with the following primary antibodies: Goat anti-CD26 pAb (Cat. No. AF1180, R&D Systems, Minneapolis, MN, USA) (1:100) and mouse anti-Ki 67 mAb (MIB-1, NB600-1252, Novus Biologicals, Littleton, CO, USA) (1:100). As the toxicity assay, the mean body weight of the mice of each group at sacrifice was measured and histological observation of hematoxylin and eosin stained samples of organs (the brain, heart, lung, liver, spleen, kidney, pancreas, digestive organs or adrenal glands) was done.

### 4.12. Statistics

In the analysis of the real-time PCR assay, the *T*-test at the *p* = 0.05 level was carried out using the SPSS software (IBM, Armonk, NY, USA). Statistical significance between the mean tumor volumes or weights of the groups in the in vivo xenograft assay was assessed by Fisher’s protected least-square differences (PLSD) multiple comparison test. Statistical analyses were carried out using the SPSS software (IBM).

### 4.13. Study Approval

All experiments were approved by the Animal Care and Use Committee of Keio University and were performed in accordance with the institute guidelines (approval number: 9184).

## 5. Conclusions

We developed an antibody-drug conjugate (ADC, designated Y-TR1) with YS110 and an inhibitor, triptolide, for one of general transcription factors for Pol II, TFIIH, using cross-linking method. Y-TR1 revealed anti-tumor property against CD26 positive cancer cells both in vitro and in vivo. Induction of nuclear localization of CD26 by Y-TR1 promotes transcriptional repression of the POLR2A gene, furthermore the internalization of YS110-TR1 compound into the nucleus may inhibit TFIIH, resulting in impaired cancer cell growth. 

## 6. Patents

Patent No. PCT/JP2016/076542 cancer treatment composition combining the anti-CD26 antibody and other anticancer agent.

## Figures and Tables

**Table 1:** IC50 of Triptolide, TR1 and Y-TR1 against MM and leukemia cell lines.

Cell Line	Origin	CD26	IC50 of Triptolide (nM)	IC50 of TR1 (nM)	IC50 of Y-TR1 (μg/mL)
MSTO wt	Mesothelioma	(-)	10	250	35
MSTO clone12	Mesothelioma	(+)	10	250	15
JMN	Mesothelioma	(+)	15	ND	30
Jurkat (-)	Leukemia	(-)	>100	ND	>100
Jurkat CD26(+)	Leukemia	(+)	6	ND	30

**Table 2:** Comparison of IC50 of Y-TR1 using various linkers (SPDP, GMBS, SMCC).

Conjugate	IC50 (μg/mL)
Y-TR1 SPDP	35
Y-TR1 GMBS	18
Y-TR1 SMCC	15

## Supplementary Materials

The following are available online at https://www.mdpi.com/2072-6694/11/8/1138/s1, Figure S1: Influence of Fc blocking reagent (2 μg/mL) on the cytotoxicity of Y-TR1 against CD26 positive MM cell line MSTO clone12 (A) and CD26 negative counterpart MSTO wt (B) was not observed. Horizontal axis shows concentration of Y-TR1 in μg/mL. Vertical axis shows percent of control of absorbance value in WST-1 assay. Figure S2: (A) The mean body weight (g) of the mice of each group at sacrifice. The error bar indicates one standard deviation. There was no significant difference between each group. (B) Histological images (hematoxylin and eosin staining) of heart, lung, liver, and kidney of Y-TR1 treated mice and control (IgG1) mice. No pathological alterations were observed.
