# Supplementary material for: Novel Antibody-Drug Conjugate with Anti-CD26 Humanized Monoclonal Antibody and Transcription Factor IIH (TFIIH) Inhibitor, Triptolide, Inhibits Tumor Growth via Impairing mRNA Synthesis
Source: Cancers (Basel). 2019 Aug 8;11(8):1138. doi: 10.3390/cancers11081138 (PMC6721810; doi:10.3390/cancers11081138)
Supplement: Supplementary file 1 [file cancers-11-01138-s001.pdf]

Supplemantry Materials: Novel antibody-drug conjugate with anti-CD26 humanized monoclonal antibody and transcription factor IIH (TFIIH) inhibitor, triptolide, inhibits tumor growth via impairing mRNA synthesis

Mutsumi Hayashi, Hiroko Madokoro, Koji Yamada, Hiroko Nishida, Chikao Morimoto, Michiie Sakamoto, Hiroshi Yanagawa and Taketo Yamada

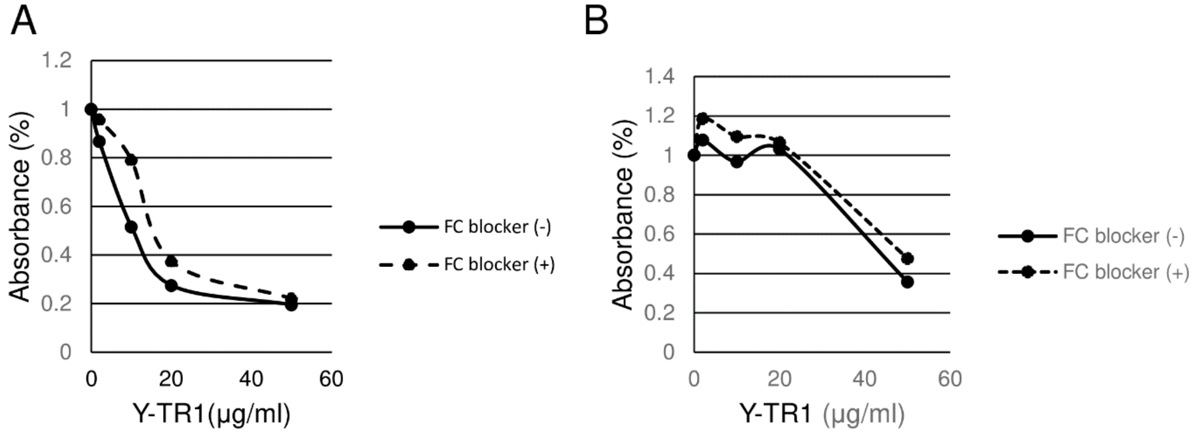

Figure S1. Influence of Fc blocking reagent (2µg/mL) on the cytotoxicity of Y-TR1 against CD26 positive MM cell line MSTO clone12 (**A**) and CD26 negative counterpart MSTO wt (**B**) was not observed. Horizontal axis shows concentration of Y-TR1 in µg/ml. Vertical axis shows percent of control of absorbance value in WST-1 assay.

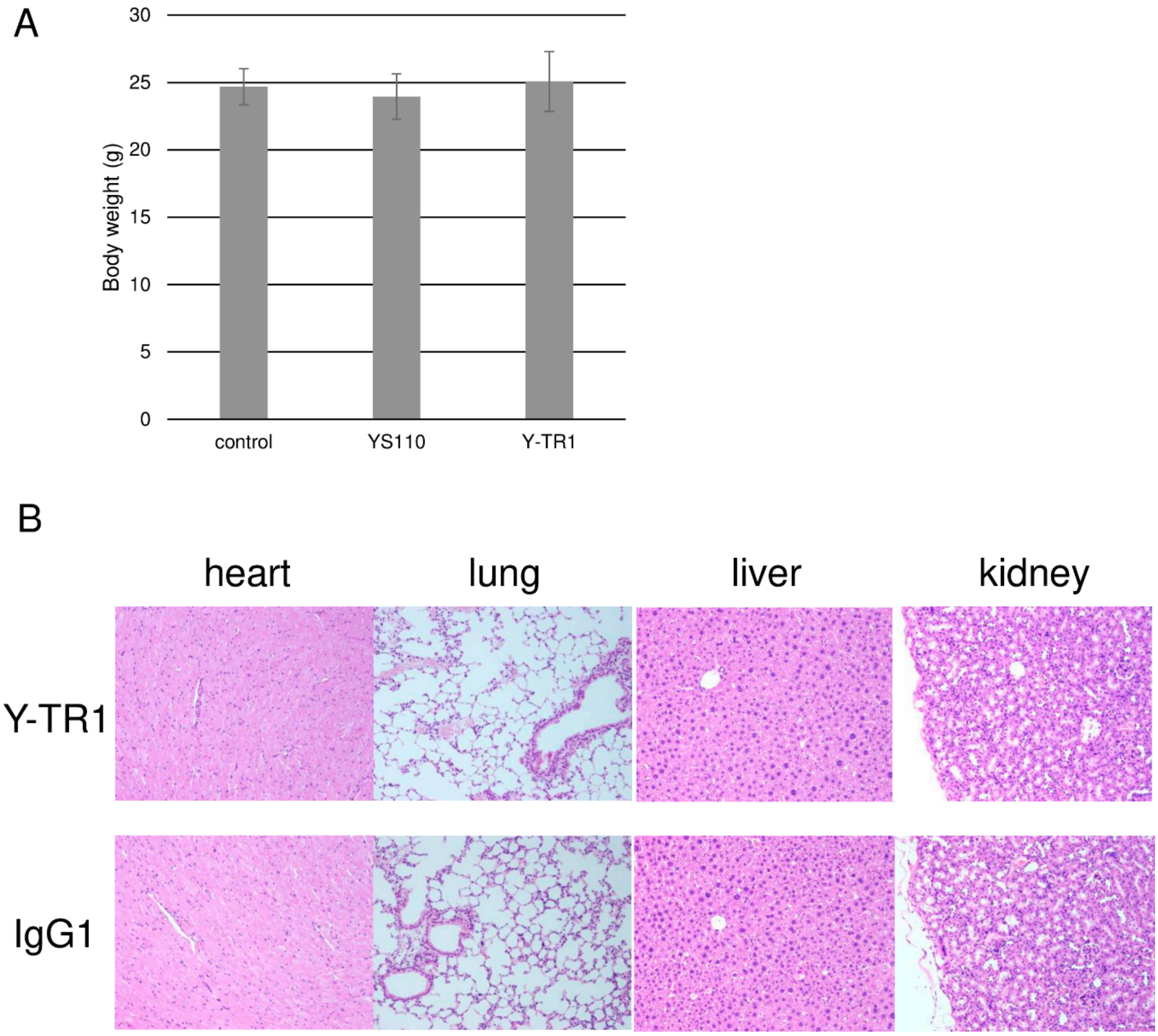

Figure S2. (**A**) The mean body weight (g) of the mice of each group at sacrifice. The error bar indicates one standard deviation. There was no significant difference between each group. (**B**) Histological images (hematoxylin and eosin staining) of heart, lung, liver, and kidney of Y-TR1 treated mice and control (IgG1) mice. No pathological alterations were observed.
